# Brain connectivity in major depressive disorder: a precision component of treatment modalities?

**DOI:** 10.1038/s41398-023-02499-y

**Published:** 2023-06-09

**Authors:** Asude Tura, Roberto Goya-Maldonado

**Affiliations:** Laboratory of Systems Neuroscience and Imaging in Psychiatry (SNIP-Lab), Department of Psychiatry and Psychotherapy, University Medical Center Göttingen (UMG), Göttingen, Germany

**Keywords:** Depression, Predictive markers

## Abstract

Major depressive disorder (MDD) is a very prevalent mental disorder that imposes an enormous burden on individuals, society, and health care systems. Most patients benefit from commonly used treatment methods such as pharmacotherapy, psychotherapy, electroconvulsive therapy (ECT), and repetitive transcranial magnetic stimulation (rTMS). However, the clinical decision on which treatment method to use remains generally informed and the individual clinical response is difficult to predict. Most likely, a combination of neural variability and heterogeneity in MDD still impedes a full understanding of the disorder, as well as influences treatment success in many cases. With the help of neuroimaging methods like functional magnetic resonance imaging (fMRI) and diffusion tensor imaging (DTI), the brain can be understood as a modular set of functional and structural networks. In recent years, many studies have investigated baseline connectivity biomarkers of treatment response and the connectivity changes after successful treatment. Here, we systematically review the literature and summarize findings from longitudinal interventional studies investigating the functional and structural connectivity in MDD. By compiling and discussing these findings, we recommend the scientific and clinical community to deepen the systematization of findings to pave the way for future systems neuroscience roadmaps that include brain connectivity parameters as a possible precision component of the clinical evaluation and therapeutic decision.

## Introduction

Major depressive disorder (MDD) is ranked as the leading cause of disability worldwide by the World Health Organization (WHO), with a lifetime prevalence of 4.4% in the general population [1]. The symptoms of MDD include depressed mood, anhedonia, i.e., diminished interest or pleasure, changes in appetite and sleep, fatigue, psychomotor agitation and retardation, feelings of worthlessness or guilt, diminished ability to concentrate, and suicidal thoughts. According to the Diagnostic and Statistical Manual of Mental Disorders-5 (DSM-5), a diagnosis of MDD requires a combination of five or more of these symptoms being present for two weeks or more, most of the day, and nearly every day [2]. Thus, the diagnosis of MDD is purely based on symptoms, and the profile of manifested symptoms may vary from patient to patient, resulting in a heterogeneous group under the same diagnostic umbrella.

Supported by evidence-based medicine, the use of more effective therapies has advanced the medical field, but insufficient clinical improvement in MDD patients still poses a challenge. The first-line treatment for MDD is pharmacotherapy and psychotherapy, with the remission rates for both modalities around 50% [3, 4]. Without knowing a priori who might benefit or not, residual depressive symptoms and disease chronification occur. Following the encouraging results presented by electroconvulsive therapy (ECT), repetitive transcranial magnetic stimulation (rTMS) has gained ground as an effective non-invasive stimulation method in the treatment of depression [5]. However, little is known about the use of neurobiological information that could potentially support more targeted clinical interventions in the future. In this context, connectivity has emerged as a very promising method for identifying and monitoring changes in the brain. Much expectation has been placed on understanding the functional, structural, or multimodal network changes associated with the alleviation of depressive symptoms. By understanding the effect of the treatment on the brain, one could improve the treatment effects, e.g., by better targeting the affected regions with a subject-tailored protocol that more precisely treat an individual presentation of MDD. Therefore, great importance is currently given to the possibility of monitoring patients with neuroimaging methods to quantify functional and structural brain networks for insights that may lead to more informed clinical decisions. However, the existing evidence is still quite fragmented.

In this review, we have assembled the evidence available on the functional and structural brain connectivity predictors of treatment response and connectivity changes after treatment, to provide a broader view and new insights of neuroimaging contibutions to depression research. We also compiled the regions and connections reported in the included studies in figures to map and compare the findings of current treatment modalities. Finally, we have offered a critical perspective on how to improve and integrate the use of functional and structural connectivity data (Box 1 and 2) to potentially inform and guide the clinical interventions as a step to crystallize systems medicine in the future.

Box 1 Measuring brain connectivity with magnetic resonance imaging **A. Functional magnetic resonance imaging**. Functional connectivity (FC) can be estimated by the temporal correlation in the blood-oxygen-level-dependent (BOLD) signal between brain regions derived from functional magnetic resonance imaging (fMRI) data [80]. Thus, when the fMRI time series between two or more regions exhibit a degree of positive correlation, it is assumed that this value quantifies the corresponding level of FC between them. When their time series show a negative degree of correlation, it is assumed that this value represents the negative level of FC, that is, these regions are anticorrelated to a certain degree. FC can be measured when the subjects are performing a task (task-based FC), or at rest, i.e., awake but not engaged in a task (task-free), the so-called resting-state functional connectivity (rsFC) [81]. RsFC can be analyzed with different methods, such as the seed-based approach, where the time series from a region of interest (ROI) is extracted and correlated with all voxels (three-dimensional volume unit of spatial resolution) of the brain [82]. Another widely used method is the independent component analysis (ICA), which is a data-driven method based on a source separation algorithm [83]. Through this method, data are divided by the algorithm into multiple components, which are interpreted as functional networks by spatiotemporal similarity according to the percentage of explained variance. Furthermore, other methods aim to understand more complex directed causal effects that are present in the brain. For this, there are effective connectivity (EC) approaches, by which the influence of one neural system on another is modeled using temporal information, for example with Granger causality analysis (GCA) [84] and dynamic causal modeling (DCM) [80].

**B. Diffusion tensor imaging**. Another type of magnetic resonance imaging protocol is called diffusion-weighted imaging (DWI). Diffusion tensor imaging (DTI) analysis can estimate the degree of diffusion of the water molecules through the white matter tracts, quantifying the spatial direction of structural connectivity (SC) underlying cortical regions [85]. Different DTI parameters such as fractional anisotropy (FA), mean diffusivity (MD), axial diffusivity (AD), and radial diffusivity (RD) can be calculated to inform about the white matter microstructure [86]. FA is a marker of axonal integrity, with lower FA values indicating weaker myelination, axonal lesion, or lower axonal density. Higher RD has been related to myelin damage, whereas lower AD has been related to axonal damage. MD is an inverse measure of membrane density, being sensitive to edema and necrosis [87]. Based on the fact that the SC supports communication between cortical regions but does not always underlie the regions displayed in FC, there is still debate about the relationship between the two connectivity methods. Nevertheless, more integrative approaches can be used for merging the structural and functional techniques for a more precise approach.

Box 2 Brain connectivity and networks in depression **A. Functional connectivity in depression**. The triple network model is proposed for the pathophysiology of psychiatric disorders, including MDD [88]. According to this model, there is aberrant FC in MDD within and between the default mode network (DMN), the frontoparietal network (FPN)—also known as the central executive network (CEN), and the salience network (SN). The DMN is extensively studied in MDD, which includes the posterior cingulate cortex (PCC), precuneus (PCu), ventromedial prefrontal cortex (vmPFC), subgenual anterior cingulate cortex (sgACC), and lateral parietal cortex [89]. The DMN is a task-negative network, meaning that its activity is increased when the subject is in the resting state, and it gets deactivated when the subject is engaged in externally-oriented tasks [82, 90]. It is important for introspection and self-referential thoughts, and its aberrant FC and neural activity has been associated with rumination and negative self-referential thoughts in MDD [88].

The FPN is activated during tasks that involve cognitive or executive functions, such as working memory, problem-solving, and decision-making [82, 91]. It primarily includes the dorsolateral prefrontal cortex (dlPFC) and posterior parietal cortex (PPC), areas that show anticorrelation with DMN during resting state and cognitive tasks, which is needed for optimized task performance [92]. Its aberrant FC and neural activity have been associated with cognitive impairment and attention deficits in MDD [88].

The SN mainly consists of the dorsal anterior cingulate cortex (dACC) and anterior insula (AI). After detecting a salient stimulus, the SN mediates the switch between FPN and DMN for externally-oriented attention or self-oriented mental processes, respectively [93]. In this way, the coordinated anticorrelation between the two networks is maintained, generating appropriate behavioral responses which can take into account the salient information, whether of primarily external or internal origin, or both. When an external salient stimulus is detected, the SN prioritizes the FPN and disengages DMN regions, directing attentional resources to external events, and activating higher cognitive processes. By prioritizing attentional resources to internal stimuli, the opposite occurs, enabling the integration of emotions, bodily sensations, and memories in decision-making. It is thought that in depressive episodes attentional resources are excessively allocated to internal self-oriented mental events at the expense of external stimuli, resulting in a tendency for negative thoughts and rumination, accompanied cognitive impairment [88].

In this context, regions of the limbic system like the amygdala and hippocampus are important for the generation of emotional states, and therefore likely play an important role in MDD [94]. Additionally, the nucleus accumbens (NAcc), ventral striatum (VS), and caudate regions are important for reward-related behavior and motivation and are probably related to anhedonia in MDD [94].

**B. Structural connectivity in depression**. According to meta-[95–97] and mega-analyses [98], there is a widespread decrease in FA in patients with MDD compared to healthy controls (HC), including in the corpus callosum (CC) [95, 97, 98], internal capsule [95], inferior longitudinal fasciculus (ILF) [97], inferior fronto-occipital fasciculus (IFOF) [97], posterior thalamic radiation [97], superior longitudinal fasciculus (SLF) [96], and corona radiata [98]. In an integrated way, it is believed that some modifications of the neural tracts identified by DTI may be behind the alterations in the FC. However, the fragmented collection of clinical and imaging information still limits the systematic evaluation of this hypothesis.

## Materials and methods

### Literature search

Studies were identified by searching with PubMed (https://pubmed.ncbi.nlm.nih.gov), Web of Science (https://www.webofscience.com), and Cochrane Library (https://www.cochranelibrary.com), using the following keywords: (depression OR major depressive disorder) AND (functional connectivity OR structural connectivity OR diffusion tensor imaging) AND (pharmacotherapy OR medication OR antidepressant OR psychotherapy OR electroconvulsive therapy OR repetitive transcranial magnetic stimulation). The search process was conducted for articles published until 19 January 2023. Parts of the figure were drawn by using pictures from Servier Medical Art. Servier Medical Art by Servier is licensed under a Creative Commons Attribution 3.0 Unported License (https://creativecommons.org/licenses/by/3.0/).

### Study selection criteria

Inclusion criteria:

- Studies that have a sample group with a MDD diagnosis
- Longitudinal studies with one of the four treatment methods: pharmacotherapy, psychotherapy, ECT or rTMS
- Studies using resting-state functional magnetic resonance imaging (rsfMRI) functional connectivity (FC) and/or diffusion tensor imaging (DTI) structural connectivity (SC) analyses

Exclusion criteria

- Other neuropsychiatric diagnoses or comorbidities (except anxiety disorders)
- Sample size smaller than 20 patients for the MDD group
- Studies that used only graph theory, network control theory, or dynamic FC analyses
- Combination of different treatment methods without detailed description
- Studies focused on elderly, adolescent, remitted, first-episode or mild-moderate MDD
- Only females or males in the sample
- Review articles, meta-analyses, letters to editors, correspondences, not full-text articles
- Full text not in English
- Additional studies using previously published imaging data from identical samples with a similar analysis

**Fig. 1:**
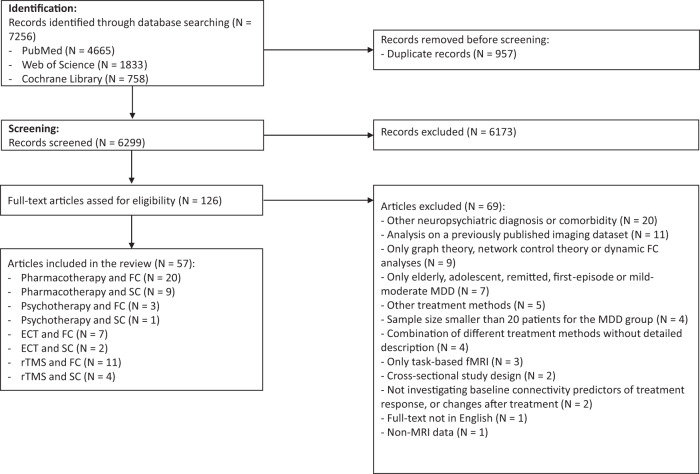
Preferred reporting items for systematic reviews and meta-analyses (PRISMA) [6] flow diagram. The numbers of identified, screened, excluded and included studies are presented.

Using the keywords, 7256 articles were identified by the search engines. After removing 957 duplicates, the remaining 6299 articles were screened by their titles and abstracts, and 6173 of them were excluded. The remaining 126 full-text articles were assessed for eligibility. Based on the selection criteria, 57 studies were included in the final review (see the PRISMA [6] flow diagram in Fig. 1).

## Results

**Fig. 2:**
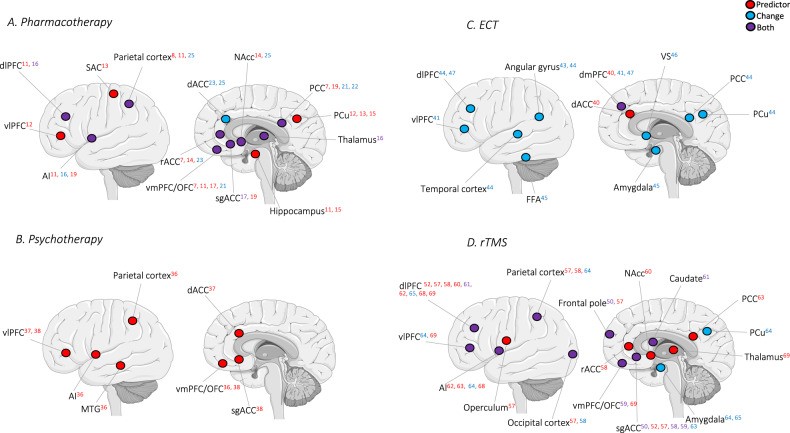
Illustrations of the main functional connectivity (FC) results from the studies included in the review. **A** Pharmacotherapy, **B** Psychotherapy, **C** Electroconvulsive therapy (ECT), and **D** Repetitive transcranial magnetic stimulation (rTMS). Color coding is red for baseline predictors of treatment response, blue for changes with treatment, and purple for predictors and changes. For simplicity, the hemisphere in the schemes does not always represent lateralization of the results.

**Fig. 3:**
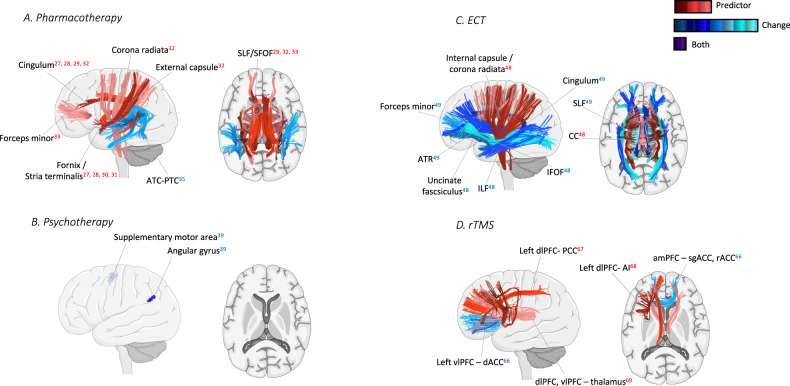
Illustrations of the main structural connectivity (SC) results from the studies included in the review. **A** Pharmacotherapy, **B** Psychotherapy, **C** Electroconvulsive therapy (ECT), and **D** Repetitive transcranial magnetic stimulation (rTMS). Tracts in shades of red represent baseline predictors of treatment response, tracts in shades of blue represent changes with treatment, and tracts in shades of purple represent predictors and changes. For simplicity, the hemisphere in the schemes does not always represent lateralization of the results.

The 57 articles included in this review (Table 1) were organized into categories (Figs. 2,3) according to the primary treatment method (A. Pharmacotherapy, B. Psychotherapy, C. ECT, D. rTMS) and connectivity data presented (1. FC, or 2. SC).

**Table 1:** Summary of findings on longitudinal studies of pharmacotherapy, psychotherapy, electroconvulsive therapy (ECT), and repetitive transcranial magnetic stimulation (rTMS).

Treatment method	Connectivity measure	Reference	Sample size	Treatment protocol	Analysis method	Main findings
Pharmacotherapy	Functional	Ang et al. [14]	241 MDD, 38 HC	-Sertraline (SSRI) or Bupropion (norepinephrine-dopamine reuptake inhibitor (NDRI)) -8 weeks -Placebo-controlled -Randomized -Double-blind	-Seed-based: NAcc -Baseline MRI	Higher baseline FC between bilateral NAcc and rACC was associated with a positive response to Bupropion but not Sertraline.
		Braund et al. [18]	226 MDD, 68 HC	-Escitalopram (SSRI), Sertraline (SSRI), or Venlafaxine-XR (SNRI) -8 weeks -Randomized	-Network-based statistic (NBS) with 436 ROIs -MRI before and after treatment	Higher baseline neuroticism was associated with greater FC within and between the SN, FPN, and SMN, which predicted poorer treatment outcomes. FC strength in these networks decreased after treatment.
		Chin Fatt et al. [9]	279 MDD, 38 HC	-Sertraline (SSRI) -8 weeks -Placebo-controlled -Randomized -Double-blind	-Whole-brain parcellation: FPN, DMN, SN, DAN, SN, SMN, hippocampus, thalamus -Baseline MRI	Higher FC within the DMN, and between the DMN and the FPN predicted better outcomes.
		Cui et al. [21]	36 MDD, 61 HC	-Escitalopram (SSRI) -12 weeks	-24 ROIs in the DMN -MRI before and after treatment	Reduced FC in the DMN core (amPFC, PCC) subsystem at baseline was increased after treatment and became comparable with that in the HC.
Pharmacotherapy (cont.)	Functional (cont.)	DeMaster et al. [11]	34 MDD	-Escitalopram (SSRI) -6 weeks	-EC: Bayesian multi-subject vector autoregressive model -Baseline MRI	Higher FC strength in DMN, FPN, and SN was related to baseline depression severity and response to treatment.
		Fischer et al. [25]	128 MDD, 58 HC	-Sertraline (SSRI), Venlafaxine-XR (SNRI), or Escitalopram (SSRI) - 8 weeks -Randomized	-Seed-based: NAcc -MRI before and after treatment	Responders had an increase in NAcc-dACC FC, which was associated with an improvement in quality of life, and a decrease in NAcc-inferior parietal lobule (IPL) FC.
		Fu et al. [17]	32 MDD, 25 HC	-Duloxetine (SNRI) -12 weeks	-ICA: DMN -MRI at weeks 0, 1, 8 and 12	Reduced baseline FC within the orbitofrontal component of the DMN predicted clinical response. There was increased FC in the anterior DMN after treatment.
		Goldstein-Piekarski et al. [7]	75 MDD, 31 HC	-Escitalopram (SSRI), Sertraline (SSRI), or Venlafaxine-XR (SNRI) -8 weeks -Randomized	-Seed-based: PCC -MRI before and after treatment	Remitters were distinguished from non-remitters by intact FC between the PCC and ACC/mPFC, similar to HC, while non-remitters had hypoconnectivity.
		Hsu et al. [16]	22 MDD, 35 HC	-Sertraline (SSRI) -6 weeks	-ICA: DMN, SN, FPN -MRI before and after treatment	Thalamo-PFC FC provided moderate predictive power for the effectiveness of treatment. FC of the medial temporal lobe of DMN, FPN, thalamus, and SN was restored to HC levels after treatment.
		Ju et al. [20]	192 MDD	-SSRIs, SNRIs, and other antidepressants -6 months	-Whole-brain parcellation with 268 ROIs -Baseline MRI	Connectome-based predictive modeling could predict symptom improvement at the 2-week, 1-, 2-, and 3-month time points after treatment.
Pharmacotherapy (cont.)	Functional (cont.)	Ju et al. [24]	110 MDD, 136 HC	-Antidepressant treatment based on clinical judgment, mostly with Paroxetine (SSRI) -6 months	-Network construction by 368 ROIs -MRI at baseline and every 6 months over 2 years	Only the DMN FC change distinguished non-remitters from the remitters at 6 months, and recurring from stable MDD during the 2-year follow-up.
		Korgaonkar et al. [10]	163 MDD, 62 HC	-Escitalopram (SSRI), Sertraline (SSRI) or Venlafaxine-XR (SNRI) -8 weeks -Randomized	-NBS with 333 ROIs -MRI before and after treatment	Remitters were distinguished from non-remitters by higher FC within the DMN. Hypoconnectivity of non-remitters distinguished them from HC at baseline and increased after treatment. FC of remitters was higher than HC at baseline and also following remission.
		Liu et al. [26]	56 MDD, 111 HC	-Paroxetine (SSRI) -6 months	-NBS -MRI before and after treatment	Impaired FC in SMN, DMN, and DAN was associated with the number of episodes and total illness duration. The disrupted FC in MDD did not change significantly after treatment.
		Martens et al. [13]	34 MDD, 31 HC	-Escitalopram (SSRI) -6 weeks	-ICA: 16 networks of interest -Baseline MRI	Treatment response was associated with increased FC of the right FPN with the posterior DMN, SMN, and somatosensory association cortex (SAC).
		van der Wijk et al. [19]	129 MDD, 99 HC	-Escitalopram (SSRI), and add-on Aripiprazole (atypical antipsychotic) for non-responders -16 weeks	-Seed-based: ACC, PCC, insula, dlPFC -Baseline MRI	Baseline FC of the ACC, PCC, and insula differentiated early-, late-, and non-remitters.
Pharmacotherapy (cont.)	Functional (cont.)	Wu et al. [12]	81 MDD	-Escitalopram (SSRI) -12 weeks	-Seed-based: 36 seeds from emotion regulation networks -Baseline MRI	Patients with remission and non-remission were separated with an accuracy of 82%. The FC between the left medial SFG, right IFG, and PCu were the features with the highest discrimination ability.
		Xiao et al. [15]	36 MDD	-Escitalopram (SSRI) -2 weeks	-Seed-based: hippocampus -Baseline MRI	The early improved patients had higher FC between the left hippocampus and left IFG and PCu than the non-improved patients, which was positively correlated with the reduction of depressive symptoms.
		Yang et al. [22]	20 MDD, 25 HC	-Sertraline (SSRI) -8 weeks	-Seed-based: PCC -MRI before and after treatment	FC of the PCC positive and PCC negative networks was lower in MDD. Treatment increased the FC in the PCC positive network, with a correlation with depression scores.
		Ye et al. [8]	66 MDD, 57 HC	-Escitalopram (SSRI) or Venlafaxine (SNRI) -4 weeks	-ICA: DMN -Baseline MRI	The remitted group had higher intra-FC in the right angular gyrus, which was positively correlated with the reduction of depressive symptoms.
		Zhang et al. [23]	59 MDD, 59 HC	-Escitalopram (SSRI), Sertraline (SSRI), or Fluoxetine (SSRI) -12 weeks	-Seed-based: ACC -MRI before and after treatment	Only a subregion of dACC and rACC had increased FC with SN after treatment.
	Structural	Davis et al. [32]	200 MDD, 112 HC	-Escitalopram (SSRI) -8 weeks	-FA, MD, AD, and RD for 40 ROIs -MRI at baseline, 2 and 8 weeks after treatment	Baseline AD in the external capsule, which overlaps the SLF, was associated with treatment response. Further baseline differences of responders compared with non-responders were in the cingulum, sagittal stratum, and corona radiata. No changes in SC were observed after the treatment.
		Dong et al. [34]	62 MDD, 118 HC	-SSRIs or SNRIs -6 months	-FA -MRI before and after treatment	Decreased FA in the left insula, left middle occipital gyrus, right thalamus, left pallidum, and left PCu were observed in MDD compared with HC. No significant change in FA was observed after the treatment.
Pharmacotherapy (cont.)	Structural (cont.)	Fan et al. [35]	59 MDD, 118 HC	-Paroxetine (SSRI) -6 months	-FA -MRI before and after treatment	Decreased SC between the right ATC and the posterior temporal cortex (PTC), and between the left temporal cortex and the auditory cortex were reversed at remission.
		Grieve et al. [27]	-74 MDD to calculate FA measures -83 MDD for replication	-Escitalopram (SSRI), Sertraline (SSRI), or Venlafaxine-XR (SNRI) -8 weeks	-FA of stria terminalis and cingulate bundle	The ratio of the FA of the stria terminalis over the cingulate identified non-remitters with an accuracy of 83-88%, with greater specificity for Escitalopram and Sertraline.
		Korgaonkar et al. [29]	157 MDD	-Escitalopram (SSRI), Sertraline (SSRI) or Venlafaxine-XR (SNRI) -8 weeks -Randomized	-FA for 46 tracts -Baseline MRI	FA in the left cingulum bundle, right SFOF, and right SLF identified 15% of the non-remitters with 84% accuracy.
		Korgaonkar et al. [28]	74 MDD, 34 HC	-Escitalopram (SSRI), Sertraline (SSRI) or Venlafaxine-XR (SNRI) -8 weeks -Randomized	-FA for cingulate, fornix, stria terminalis and uncinate fasciculus -Baseline MRI	Altered SC of the cingulate and stria terminalis predicted remission with 62% accuracy. Prediction improved to 74% when age was added to the model.
		Pillai et al. [31]	144 MDD	-Sertraline (SSRI) -Placebo-controlled	-FA between raphe nucleus, amygdala, and hippocampus -Baseline MRI	FA was lower in remitters than in non-remitters in raphe nucleus-amygdala tracts, which correlated with depressive scores.
		Vieira et al. [33]	20 MDD	-Paroxetine (SSRI) -6-12 weeks	-FA, MD, AD, RD -Baseline MRI	There was an increase in FA and a decrease in RD in forceps minor and SLF in responders compared to non-responders at baseline.
Pharmacotherapy (cont.)	Structural (cont.)	Zhou et al. [30]	40 MDD	-SSRIs -8 weeks	-FA -Baseline MRI	Reductions in FA were found in the hippocampus in treatment-resistant depression compared to MDD.
Pharmacotherapy or psychotherapy	Functional	Dunlop et al. [38]	122 MDD	-CBT (16 sessions) or pharmacotherapy (Escitalopram or Duloxetine, double-blind) -Randomized -12 weeks	-Seed-based: sgACC -Baseline MRI	FC of the left vlPFC, the dorsal midbrain, and the left vmPFC with the sgACC was differentially associated with remission to CBT and pharmacotherapy. Positive FC was associated with remission with CBT and treatment failure with pharmacotherapy, whereas negative FC was associated with remission to pharmacotherapy and treatment failure with CBT.
Psychotherapy	Functional	Crowther et al. [36]	23 MDD, 20 HC	-Behavioral activation treatment -12 sessions	-Seed-based: AI, dACC, superior parietal lobule (SPL), dlPFC, PCu, mPFC -Baseline MRI	Clinical response was predicted by baseline FC of the right AI with the right MTG, and the left intraparietal sulcus with the OFC.
		Späti et al. [37]	21 MDD, 35 HC	-CBT -22 weeks	-Seed-based: medial frontal gyrus (MFG) -MRI at baseline	Increased FC of PFC with the dACC was related to higher levels of adaptive rumination and better response to treatment.
	Structural	Wang et al. [39]	21 MDD, 22 HC	-Guided imagery psychotherapy -4 weeks	-FA -MRI before and after treatment	MDD had increased FA in the right thalamus compared to HC. SC changes did not recover after treatment, but a novel region of increased FA was found in the supplementary motor area.
ECT	Functional	Leaver et al. [46]	33 MDD, 33 HC	-Right unilateral or a combination of right unilateral and bifrontal ECT -11.8(±3.3) sessions -2–4 weeks	-ICA: DMN, SN, thalamus/basal ganglia network -MRI at baseline and after treatment	VS-ventral DMN hyperconnectivity was reduced, while VS-anterior DMN hypoconnectivity only modestly improved after treatment. FC between the SN and dmPFC was reduced in MDD, but did not change after treatment.
ECT (cont.)	Functional (cont.)	Mo et al. [44]	28 MDD, 20 HC	-Modified bifrontal ECT	-Seed-based: Left angular gyrus -MRI at baseline	FC of the left angular gyrus with bilateral inferior temporal gyrus, bilateral MFG, left SFG, left MTG, left PCu, left PCC, and right angular gyrus was strengthened after treatment.
		Pang et al. [41]	33 MDD	-Modified bifrontal ECT -7.49 (±1.92) sessions	-Seed-based: mPFC, PCu, angular gyrus, parahippocampus, MTG, vlPFC, dlPFC, precentral gyrus, cerebellum -MRI before and after treatment	Baseline FC within the DMN, and between the DMN and FPN could predict clinical improvement. FC within the DMN and between DMN and FPN increased after treatment, and the changed FC between the dmPFC and vlPFC was negatively correlated with clinical improvement.
		van Waarde et al. [40]	45 MDD	-Right unilateral or bilateral ECT -18.7(±7.1) sessions	-ICA -Baseline MRI	Networks centered in the dmPFC and the ACC had a sensitivity of 80–84% and a specificity of 75–85% for predicting recovery.
		Wang et al. [45]	23 MDD, 25 HC	-Modified bifrontal ECT -three times a week -2–3 weeks	-Seed-Based amygdala -GCA: the amygdala and fusiform face area (FFA) -MRI before and after treatment	There was increased FC between left amygdala and left FFA, and EC from FFA to the amygdala after treatment.
		Wei et al. [43]	26 MDD, 20 HC	-Modified bifrontal ECT -3 times a week until remission	-Whole-brain connectivity matrix -MRI before and after treatment	FC strength of the left angular gyrus increased after treatment.
		Zhang et al. [47]	46 MDD, 33 HC	-Modified bifrontal ECT -6–12 sessions	-Seed-based: dmPFC, dlPFC, OFC -MRI at baseline and after treatment	There was a correlation between the changed dmPFC-dlPFC FC after treatment and the change in anhedonia.
ECT (cont.)	Structural	Lyden et al. [49]	20 MDD, 28 HC	-Right unilateral, or right unilateral and bitemporal ECT	-FA, RD, AD, MD -MRI before the first ECT, after the second ECT, and after completion of the index ECT series	There was increased FA in the anterior cingulum, forceps minor, and left SLF after treatment. There were decreases in RD and MD in overlapping regions and the ATR. Changed SC in pathways connecting frontal and limbic areas was related to therapeutic response.
		Repple et al. [48]	98 MDD, 52 HC	-ECT -13.86(±3.53 sessions)	-FA, MD, RD, AD -MRI before and after treatment	Baseline FA was positively and MD negatively correlated with symptoms after ECT, mainly in CC, internal capsule, and corona radiata. MD increased after ECT in the right uncinate fasciculus, posterior limb of the internal capsule, ILF, and IFOF.
rTMS	Functional	Baeken et al. [50]	20 MDD	-20 Hz rTMS -20 sessions -To the left dlPFC -Sham-controlled	-Seed-based: sgACC -MRI before and after treatment	Responders had stronger negative FC between the sgACC and left dmPFC. After successful treatment, there was stronger positive FC in these regions.
		Baeken et al. [59]	44 MDD, 44 HC	-Accelerated intermittent theta burst stimulation (aiTBS) -To the left dlPFC -20 sessions -Sham-controlled	-Seed-based: sgACC -MRI before and after treatment	A positive sgACC FC with the mOFC could distinguish responders from non-responders at baseline. Treatment increased sgACC–mOFC FC, which was associated with a decrease in feelings of hopelessness.
		Chen et al. [64]	20 MDD, 20 HC	-10 Hz rTMS -To the left dlPFC -25 sessions -4 weeks -Sham-controlled	-Seed-based: amygdala -MRI before and after treatment	There was an increase in FC in the left insula, right IFG, right SFG, and right IPL; and a reduction in FC in the PCu after treatment. Change in FC between the left insula and left amygdala was positively correlated with the change in depressive scores.
rTMS (cont.)	Functional (cont.)	Du et al. [60]	22 MDD	-High frequency (HF) rTMS -To the left dlPFC -10 sessions -2 weeks	Seed-based: Left dlPFC stimulation target, left NAcc -Baseline MRI	The early improvers had increased negative FC between the stimulated dlPFC and left NAcc compared to non-improvers. The stimulated dlPFC–NAcc FC negatively correlated with improved depressive symptoms.
		Eshel et al. [65]	36 MDD, 28 HC	-10 Hz rTMS -To the left dlPFC -20 sessions -Randomized -Sham-controlled -Double-blind	-Seed-based: Left dlPFC stimulation target -MRI before and after treatment	There was an impaired inhibitory effect of the dlPFC on the amygdala in MDD compared to HC. rTMS increased dlPFC global FC and induced a negative dlPFC-amygdala FC.
		Ge et al. [58]	32 MDD, 24 HC	-10 Hz rTMS or iTBS -To the left dlPFC -20-30 sessions	-Seed-based: sgACC, rACC -MRI before and 12 weeks after the treatment	Treatment response was associated with lower FC of sgACC to right dlPFC, and higher FC of rACC to left lateral parietal cortex at baseline. The hyperconnectivity between sgACC and the visual cortex was normalized to a level comparable to that of HC after treatment.
		Iwabuchi et al. [62]	27 MDD	-10 Hz rTMS or iTBS -16 sessions -4 weeks -To the left dlPFC	-Seed-based: right AI -GCA: right AI and left dlPFC stimulation target -ICA: DMN, CEN, SN. -MRI before and after treatment	Baseline fronto-insular EC and SN FC were positively correlated with early (1 month) response to treatment but not sustained response (3 months). FC measures did not change significantly after treatment.
		Kang et al. [61]	24 MDD	-10 Hz rTMS -To the left dlPFC -10 sessions -2 weeks -Sham-controlled	Seed-based: dlPFC -MRI before and after treatment	Reduced dlPFC-left caudate FC predicted clinical improvement. There was a reduction of the FC between these regions after treatment compared to sham.
		Hopman et al. [57]	70 MDD	-HF rTMS -To the left dlPFC -20 sessions -4 weeks	-Seed-based: Left dlPFC, sgACC -Baseline MRI	dlPFC-sgACC FC was not associated with treatment outcome. Long-term non-responders had poorer FC between the sgACC-frontal pole, SPL, and occipital cortex, and dlPFC-central opercular cortex.
		Taylor et al. [63]	32 MDD	-10 Hz rTMS -To the left dlPFC -20 sessions -Sham-controlled	-Seed-based: sgACC, amygdala, PCC, dlPFC stimulation site -MRI before and after treatment	Baseline FC between PCC and AI was lower in responders compared to non-responders. There was no significant effect of rTMS over sham on the depressive scores or on FC. sgACC FC to AN, DMN, and FPN decreased in responders, but not in non-responders.
rTMS (cont.)	Functional (cont.)	Weigand et al. [52]	41 MDD	-rTMS -To the left dlPFC -28.5(±3.4) sessions -4–7 weeks -Sham-controlled	Seed-based: left dlPFC stimulation target and sgACC -Baseline MRI	Treatment efficacy was predicted by left dlPFC stimulation sites that were more anterolateral and more negatively correlated with the sgACC.
	Structural	Klooster et al. [67]	40 MDD	-aiTBS -To the left dlPFC -5 sessions -4 days -Sham-controlled	- FA, MD, tract density, tract volume, and number of tracts: between left dlPFC and ACC -Baseline MRI	SC between the patient-specific left dlPFC stimulation site and the PCC had the predictive potential for clinical response.
		Ning et al. [66]	21 MDD	-10 Hz rTMS -Left dlPFC -36 sessions	- FA, RD, AD: left PFC -MRI before and after treatment	Baseline SC of the dACC and vlPFC were correlated with changes in depressive scores. FA was increased, and RD was decreased in amPFC. SC changes in the vlPFC were correlated with treatment response.
rTMS (cont.)	Functional and Structural	Chen et al. [69]	33 MDD	-rTMS -To the left dlPFC -10 sessions	-Seed-based: PFC, motor, somatosensory, parietal, and temporal cortices, thalamus -FA: thalamocortical tracts -Baseline MRI	FC and SC between the thalamus (mediodorsal nucleus) and PFC (dlPFC, vlPFC, OFC) predicted treatment efficacy.
		Fu et al. [68]	27 MDD	-rTMS -To the left dlPFC -10 sessions -2 weeks	Seed-based: left dlPFC and bilateral insula -FA: tracts between left dlPFC and bilateral insula -Baseline MRI	FC and SC between left dlPFC and insula had a positive correlation with clinical improvement.

### Pharmacotherapy and functional connectivity

Of the 13 studies that primarily focused on the association between baseline FC and response to selective serotonin reuptake inhibitors (SSRIs) and serotonin-norepinephrine reuptake inhibitors (SNRIs), 11 studies reported increased FC within the brain that was associated with better treatment outcomes. Most of these results converged on brain regions that are part of the default mode network (DMN) as a potential biomarker of treatment response. Goldstein-Piekarski et al. (2018) reported that remitters had intact FC between the anterior and posterior parts of the DMN, compared to the hypoconnectivity seen in the non-remitters to SSRIs and SNRIs [7]. Ye et al. (2022) reported increased baseline right angular gyrus FC within the DMN in the remitted group compared with the non-remitted group, which was positively correlated with the reduction of depressive scores [8]. In addition to the increased FC within the DMN that was associated with better treatment outcomes [9–11], increased FC between DMN and frontoparietal network (FPN) [9, 10, 12], and somatomotor network (SMN) [10] was also reported. Additionally, treatment response was associated with increased FC between the right FPN and the posterior DMN, SMN, and somatosensory association cortex (SAC) by Martens et al. [13]. Increased FC between bilateral nucleus accumbens (NAcc) and rostral anterior cingulate cortex (rACC) was also associated with better treatment outcomes by Ang et al. (2020), but this effect was specific to Bupropion but not Sertraline [14]. Increased FC in other regions that were associated with better treatment outcomes was also reported, e.g., between the left hippocampus and left inferior frontal gyrus (IFG), and precuneus (PCu) [15]; and between left superior frontal gyrus (SFG) and thalamus [16]. Contrary to the aforementioned results, two studies reported decreased FC associated with better treatment outcomes, one within the orbitofrontal part of the DMN, including the subgenual anterior cingulate cortex (sgACC) [17], and one within and between the salience network (SN), FPN, and SMN [18]. Finally, van der Wijk et al. (2022) reported a mixed effect, i.e., higher FC in the posterior DMN and lower FC in the anterior DMN in early remitters compared to late- and non-remitter MDD [19]. In addition to these results that are limited to relatively short-term follow-ups, Ju et al. (2020) reported that connectome-based modeling could predict clinical improvement at 2 weeks, 1, 2, and 3 months after treatment [20].

Similar to the results for the biomarkers of treatment response, the change in FC with pharmacotherapy is still inconclusive, but the emphasis on the DMN is again evident. Cui et al. (2021) reported that the reduced FC in the anteromedial prefrontal cortex (amPFC) and posterior cingulate cortex (PCC) within the DMN was increased after treatment to levels comparable to those in the healthy controls (HC) [21]. In line with this, Yang et al., (2016) reported increased FC strength in the posterior DMN, including PCC, after treatment with Sertraline [22]. Fu et al. (2015) also found increased DMN FC after treatment, but only in the anterior components [17]. These results might suggest that DMN subnetworks can be affected differently by pharmacotherapy. This view is supported by Hsu et al. (2021), reporting that the FC between the medial temporal part of DMN and FPN was normalized after pharmacotherapy, while the core part of the DMN remained impaired [16]. The findings from Zhang et al. (2023) also support the idea that subnetworks of regions can be affected differently by treatment. They reported that only a subregion of the dorsal anterior cingulate (dACC) and rACC had increased FC with SN after treatment with SSRIs, while the FC of two other subregions of the anterior cingulate cortex (ACC) did not change after treatment [23]. Further emphasizing the importance of the DMN for the effect of pharmacotherapy on brain coupling, Ju et al. (2022) showed that only the decrease in DMN FC after pharmacotherapy distinguished remitters from non-remitters at 6 months, and stable from recurrent MDD during the 2-year follow-up [24]. In addition to the DMN, the reward system also appears to be important for the effect of pharmacotherapy, since increased FC between NAcc and dACC after treatment was associated with an improved physical quality of life [25]. Other networks were also reported to have decreased FC after pharmacotherapy, including SN, FPN, and SMN [18]. Contrary to these findings, Liu et al., (2021) reported that impaired FC in SMN, DMN, and dorsal attention network (DAN) in MDD was associated with the number of episodes and total illness duration, but FC did not change significantly when the patients achieved remission with Paroxetine [26].

### Pharmacotherapy and structural connectivity

Of the seven studies that investigated the baseline SC as potential biomarkers of response to SSRIs and SNRIs, five of them focused only on the fractional anisotropy (FA) parameter. Three studies made predictions about patients’ clinical responses based on baseline FA parameters, reaching relatively high accuracies (62–88%). One of them predicted the status of remission with baseline FA in stria terminalis [27], another with stria terminalis and cingulum [28], and a third study with cingulum, superior fronto-occipital fasciculus (SFOF), and superior longitudinal fasciculus (SLF) [29]. Moreover, Zhou et al. (2011) found higher FA in the white matter tracts of the hippocampus bilaterally in treatment responders compared to non-responders at baseline [30]. Contrary to this finding, as well as to the authors’ hypotheses, Pillai et al. (2019) found lower FA in responders in the tracts between the raphe nucleus and amygdala compared to non-responders [31]. In addition to these studies focusing only on the FA parameter, two studies investigated four DTI parameters: Davis et al. (2019) reported that axial diffusivity (AD) in the external capsule and SLF was associated with clinical response [32], and Vieira et al. (2021) reported that higher FA and lower radial diffusivity (RD) in forceps minor and SLF was evident in responders compared to non-responders to Paroxetine [33].

Davis et al. (2019) and Dong et al. (2020) investigated the effect of SSRIs and SNRIs on SC but found no significant changes after 8 weeks, and 6 months after treatment, respectively [32, 34]. Fan et al. (2020) found four SC alterations in patients with MDD compared to HC. After 6 months of Paroxetine, two of these alterations, between the right anterior and posterior temporal cortex, and between the left temporal cortex and the auditory cortex, reversed at remission, hinting towards state-dependent alterations in MDD [35].

### Psychotherapy and functional connectivity

Although studies that investigated the relationship between brain connectivity and psychotherapy are scarce, Crowther et al. (2015) reported that the FC between the right insula and right middle temporal gyrus (MTG), and left intraparietal sulcus and orbitofrontal cortex (OFC) predicted response to behavioral activation therapy [36]. In addition, Späti et al. (2015) reported that higher FC between the frontal pole and dACC was related to higher levels of adaptive rumination and better response to cognitive behavioral therapy (CBT) [37]. The study by Dunlop et al., (2017), in which MDD patients were randomized to either CBT, Escitalopram, or Duloxetine, found that positive FC of sgACC with left ventrolateral prefrontal cortex (vlPFC), left ventromedial prefrontal cortex (vmPFC) and dorsal midbrain was associated with remission with CBT [38]. Conversely, negative FC of the same regions was associated with remission due to pharmacotherapy, suggesting different biomarkers of treatment response for different treatment methods.

### Psychotherapy and structural connectivity

Wang et al. (2013) reported that the increased FA in the right thalamus in patients with MDD compared to HC did not change after guided imagery psychotherapy, but novel regions of increased FA were found in the left frontal lobe (supplementary motor area), and a decreased FA in the right angular gyrus white matter after the treatment [39]. Hence, psychotherapy might be exerting its clinical effects by changing the white matter integrity in additional regions, rather than recovering the aberrant SC profile evident in MDD. However, more studies are needed to confirm this hypothesis.

### Electroconvulsive therapy and functional connectivity

Van der Waarde et al. (2015) focused on the predictors of ECT treatment response based on FC measures and reported that networks centered in the dorsomedial prefrontal cortex (dmPFC) and ACC could predict recovery with relatively high sensitivity (80 and 84%) and specificity (75 and 85%) [40]. According to Pang et al. (2022), baseline FC within the DMN and between the DMN and FPN could effectively predict the improvement of symptoms after ECT [41]. Contrary to these results, Chen et al. (2017) reported that the baseline FC was unrelated to the treatment response after ECT in their MDD patient sample [42].

Wei et al. (2018) and Mo et al. (2020) reported that the FC strength of the left angular gyrus increased after ECT [43, 44]. But in both of the studies, there was no difference in left angular gyrus FC between HC and patients with MDD in the baseline. Interestingly, the increased FC of the angular gyrus after ECT returned to HC levels one month after ECT [43]. Thus, the increased FC of the left angular gyrus in MDD seems to temporarily deviate from the HC levels with the intervention, eventually returning to normal levels after about a month. Wang et al. (2017) reported that there was increased FC between the left amygdala and left fusiform face area (FFA), and increased effective connectivity (EC) from FFA to the amygdala after ECT, which became closer to HC levels [45]. In line with this, Leaver et al. (2016) reported that the hyperconnectivity between ventral striatum (VS) and ventral DMN returned to normal levels after ECT, and the hypoconnectivity between the VS and anterior DMN modestly improved. However, the FC between the dmPFC and SN remained impaired after ECT [46]. Additionally, two studies found an association between change in FC after ECT and change in clinical measures. Pang et al. (2022) reported that the change in the FC between the medial prefrontal cortex (mPFC) and vlPFC was negatively correlated with symptom improvement [41]. Very similarly, Zhang et al. (2021) reported that the changed FC between dmPFC and dlPFC after ECT was associated with the amelioration of anhedonia [47].

### Electroconvulsive therapy and structural connectivity

Although studies investigating baseline predictors of ECT response are very scarce, Repple et al. (2020) reported that baseline FA and mean diffusivity (MD), mainly in the corpus callosum (CC), internal capsule, and corona radiata, were positively and negatively associated with symptoms post-ECT, respectively [48].

Studies investigating changes in SC after ECT have also been scarce and their results are inconsistent. Lyden et al. (2014) found a decrease in RD and MD in the cingulum, forceps minor, left SLF, and anterior thalamic radiation (ATR), as well as increased FA in these tracts after ECT. The changes in the SC parameters of MDD were correlated with changes in the severity of depression, and were in the direction of HC values, suggesting a “normalization” [49]. Differently, Repple et al. (2020) found an increase in MD after ECT in the right hemisphere, mainly in the uncinate fasciculus, posterior limb of the internal capsule, inferior longitudinal fasciculus (ILF) and inferior fronto-occipital fasciculus (IFOF). But these changes deviated from HC values, and according to the authors, this discrepancy possibly reflects increased permeability of the blood-brain barrier after ECT, resulting in disturbed communication of fibers [48].

### Repetitive transcranial magnetic stimulation and functional connectivity

Much emphasis has been placed on the FC of sgACC as a potential predictor of rTMS treatment response. Baeken et al. (2014) reported that responders showed stronger anticorrelation between the sgACC and left dmPFC compared to non-responders [50], in line with the idea that clinical efficacy of rTMS related to sgACC-dlPFC anticorrelation [51]. Later, Weigand et al. (2018) also showed that rTMS efficacy was predicted by dlPFC stimulation sites that were more anterolateral and negatively correlated with sgACC [52], which is later replicated by further studies [53–56]. Contrary to these results, Hopman et al., (2021) did not evidence that baseline left dlPFC-sgACC FC was associated with treatment outcome [57]. Alternatively, Ge et al. (2020) reported that higher sgACC-dlPFC negative FC was associated with better response, but only on the right dlPFC, while the stimulation was delivered on the left dlPFC. The association with better rTMS treatment response was also evident in the rACC and left lateral parietal cortex [58]. In addition, Baeken et al. (2017) reported that sgACC FC with medial orbitofrontal cortex (mOFC) could distinguish responders and non-responders to rTMS treatment [59]. Furthermore, FC between the stimulation site in the dlPFC and other regions has also been reported to be associated with rTMS treatment response: Du et al. (2017) reported that increased negative FC between the stimulated dlPFC site and left NAcc could distinguish early improvers compared to non-improvers, and the FC strength negatively correlated with clinical efficacy [60]. Moreover, Kang et al. (2016) reported that decreased FC between the dlPFC and left caudate predicted clinical improvement after rTMS treatment [61]. Another region that was reported to predict rTMS treatment response was the anterior insula (AI). Iwabuchi et al. (2019) reported that fronto-insular EC and SN FC were positively correlated with early (1 month), but not sustained response (3 months) to rTMS [62]. Besides, Taylor et al. (2018) reported that FC between AI and PCC was lower in responders compared to non-responder to rTMS [63].

The change in the FC of the sgACC after rTMS treatment has also been studied extensively. Baeken et al. (2014) reported that the anticorrelated FC between the sgACC and the dmPFC was stronger in responders compared to non-responders at baseline. But after rTMS treatment, a positive correlation in FC between these regions was seen in responders compared to non-responders [50]. Later, Baeken et al. (2017) reported that baseline FC between sgACC and mOFC could distinguish responders and non-responders. They also reported that the FC between these regions increased after effective rTMS treatment, which was associated with a decrease in feelings of hopelessness [59]. Furthermore, Ge et al. (2020) reported that the hyperconnectivity between sgACC and visual cortex “normalized” after rTMS treatment [58]. Conversely, Iwabuchi et al. (2019) and Taylor et al. (2018) did not find any significant change in the FC profile of patients with MDD after active rTMS treatment compared to sham [62, 63]. But Taylor et al. (2018) further stated that sgACC FC to AN, DMN, and FPN decreased among patients who showed significant improvement after rTMS treatment, but not in non-responders [63], suggesting that such FC changes might be specific to the patients who show clinical improvement. Furthermore, Kang et al. (2016) reported that there was a greater reduction of FC strength between the dlPFC and left caudate after active rTMS compared to sham [61]. Change in the amygdala FC has also been reported by two studies. Chen et al. (2020) reported that the degree of “normalization” in FC between the left insula and amygdala was correlated with a change in depressive scores [64]. Finally, Eshel et al. (2020) showed that rTMS induced negative dlPFC-amygdala FC towards normative values, such that the dlPFC was better able to engage in top-down control of the amygdala [65].

### Repetitive transcranial magnetic stimulation and structural connectivity

Studies investigating the relationship between rTMS and SC mainly focused on the connectivity of the left dlPFC stimulation site. Ning et al. (2022) reported that baseline SC of the vlPFC and dACC was correlated with changes in depressive scores [66]. Furthermore, Klooster et al. (2020) also reported that the indirect SC between the patient-specific stimulation site on the left dlPFC and the cingulate cortex has predictive potential for clinical response to rTMS treatment [67]. Two studies investigated functional as well as structural connectivity predictors of rTMS response: Fu et al. (2021) reported that functional and structural connectivity between the left dlPFC and insula [68], and Chen et al. (2022) reported that thalamo-prefrontal functional and structural connectivity predicted the efficacy of rTMS [69].

Ning et al. (2022) did not find any significant changes in the tracts connected to the stimulation targets on the left dlPFC after rTMS, but they found that rTMS increased FA and decreased RD in anteromedial prefrontal fiber bundles. The authors also report that the changes in the lateral prefrontal white matter tracts were significantly correlated with treatment response [66].

## Discussion

In this article, we reviewed 57 longitudinal studies that investigated the functional and structural connectivity in patients with MDD undergoing four different treatment methods: Pharmacotherapy, Psychotherapy, ECT, and rTMS. We summarized the findings of the included studies for each treatment method (Table 1). Additionally, we displayed these findings on brain models (Figs. 2,3) to help visualize and compare the evidenced relationship between different treatment methods, and functional and structural connectivity. Finally, we made recommendations that seem crucial to us to overcome the limitations that still exist in this area.

For FC, we identified some regions that are commonly associated with different treatment methods. For instance, dlPFC and ACC seem to be common regions associated with pharmacotherapy [7, 12, 14, 15, 19, 23, 25, 70], as well as rTMS [50, 52, 57–65, 71]. Additionally, PCC has been reported more often in pharmacotherapy studies than in studies with other treatment methods [7, 19, 21, 22]. Noteworthy, the use of different classes of antidepressants in different studies might have caused variability in the results, which the available data do not allow us to evaluate with certainty. Compared to other treatment methods, dmPFC has been more frequently reported in ECT studies [40, 41, 47]. There are fewer studies on psychotherapy, but vlPFC and vmPFC have been commonly reported in the studies that used this treatment method [36–38]. Overall, the association of higher anticorrelation between the left dlPFC and the sgACC and better clinical efficacy after rTMS has been the finding that was replicated the most [54–60]. These promising findings support the personalization of rTMS stimulation sites on the left dlPFC based on patient-specific functional magnetic resonance imaging (fMRI) data using neuronavigation, with higher clinical success [72, 73]. A particularly promising aspect of rTMS, due to the stimulation focality of the figure-of-eight coil, is the possibility of personalizing treatment according to particular circuits implicated in symptoms manifested by the patient. The underlying functional and structural connections between the stimulation target, and the regions which may be directly implicated in different symptoms, such as psychomotor retardation, cognitive impairment, lack of emotion regulation, or somatic symptoms, can be useful. For instance, by using the connectivity information, the regions and tracts associated with the particular symptoms can possibly be modulated according to the symptom profile of the patient. Nevertheless, more prospective studies are needed to establish the validity of this precision approach.

For SC, results were vastly different for different treatment methods. In pharmacotherapy, cingulum [29, 32], forceps minor [33], SLF [29, 32, 33], SFOF [29], and white matter tracts that connect the limbic regions [27, 28, 30, 31] have been commonly reported as possible baseline predictors of treatment response. In the two rTMS studies, analysis of SC has been performed with seed-based methods, where the region of interest (ROI) selected was the stimulation target on the left dlPFC [66, 67]. This likely converged the results to particular white matter tracts that connect dlPFC to other regions. Nevertheless, considering the possibility of long-distance effects as suggested in FC, this may result from stronger focal intervention in rTMS in contrast to the effects of the other more systemic interventions. The results from the two ECT studies were widespread, but inconsistent, which makes it hard to make conclusions [48, 49]. Lastly, there is only one study investigating the effect of psychotherapy on SC, reporting results in frontal and parietal regions [39], which reinforces the need for more studies investigating this topic.

As the summary suggests, although there seems to be common regions reported in different FC and SC studies, the results remain quite inconsistent. To overcome this reproducibility problem and the difficulty of directly comparing results from different treatment modalities, we propose recommendations for future studies:

1. **Standardization of the analysis methods**. The lack of standardization in the analysis methods hinders the comparability of the effects of different treatment modalities. For example, the choice of different seeds as ROI within or across different treatment modalities prevents the comparison between studies. In general, the selection of ROIs in a study invariably produces partial connectivity results, and such regions may not even be selected for evaluation in a second study. Therefore, it becomes impossible to compare studies if the raw data are not shared by the authors.
2. **Larger sample sizes for single-site studies and data sharing for multicenter analyses**. One of the main reasons behind high variability and low reproducibility in the results is most likely the limited sample size, hence low statistical power and higher rates of false positives. When this aspect is combined with the heterogeneity in the MDD samples, it makes it very difficult to compare the effect of different treatment modalities or to conclude what the effect of each treatment method is. According to statistical power analyses; for a liberal threshold of 0.05, ~12 subjects are required to achieve 80% power at the single voxel level, and double the number of subjects are needed to maintain this level of power at more realistic thresholds correcting for multiple comparisons [74]. According to an evaluation, highly cited clinical fMRI studies published in high-impact journals between 1999–2018 had a median sample size of 14.5 subjects, which increased at a rate of 0.74 participant/year. Furthermore, only 9 out of 273 papers published in 2017 and 2018 had pre-study power calculations [75], suggesting that most neuroimaging studies might not have enough statistical power. This can contribute to the reporting of false positives and the reproducibility problem. Alarmingly, a recent article stated that reproducible brain-wide association studies (BWAS), studies investigating the associations between brain function or structure and complex cognitive or mental health phenotypes, require samples with thousands of individuals [76]. Therefore, we intend to raise the attention to the clinical and scientific communities to the importance of bigger sample sizes, pre-study power calculations, and sharing of clinical and imaging data of patients with MDD, which will allow more representative descriptions of the disorder on a global scale. This way, large-scale multicenter datasets analyzed via meta- and mega-analytical approaches can fill the existing gaps of knowledge about treatment modalities and related functional and/or structural connectivity.
3. **Longitudinal rather than cross-sectional study designs**. Although longitudinal studies are harder to conduct due to higher costs, time, and drop-out rates, their importance was highlighted by the article that stated the importance of larger sample sizes for BWAS studies. They stated that: “For greater effect sizes and statistical power, neuroscience should focus on within-participant study designs over cross-sectional study designs, and on interventional (therapy, medications, brain stimulation, and surgery) over observational study designs” [76]. Especially, there is a lack of long-term follow-up of patients with neuroimaging. To our knowledge, there is only one long-term study where they followed up patients on pharmacotherapy every 6 months for 2 years with magnetic resonance imaging (MRI) [24]. These data are important to understand how long these connectivity changes may last after treatment, to show whether their patterns return to the diseased state over time and whether these patterns may be directly related to relapses. This feature becomes fundamental due to the high rate of recurrence of the disorder. In the future, this information could help in clinical decisions with more difficult cases, even before the constitution of an evident relapse is fully manifested by symptoms.
4. **Randomized studies comparing different treatment methods**. These types of studies are needed for the validation of baseline biomarkers of response to specific treatment methods. Such studies are scarce, with only two comparative studies [38, 48] included in the review. Randomized studies like these can be very informative, aiming to help clinicians in their treatment choices in the future, stratifying the patient with the aid of neurobiological data.
5. **Analysis of multimodal data**. Finally, we want to emphasize the importance of multimodal imaging for a more holistic understanding of the pathophysiology of MDD and the effects of its treatment. A coordinate-based meta-analysis of neuroimaging studies that examined the multimodal brain abnormalities in MDD identified spatially convergent structural and functional abnormalities in the sgACC, hippocampus, amygdala, and putamen [77], hinting towards commonalities in the functional and structural connectivity profiles, and the importance of multimodal studies in MDD research. Other promising approaches, such as structural covariance networks [78, 79], can add more layers of information about the organization of large-scale brain networks, and, therefore, should be incorporated in future analyses.

In conclusion, to overcome the reproducibility problem and to conduct studies and analyses that would be impactful for the patient, scientific and clinical communities, we reinforce the need of standardizing and systematizing the collection, curation, analysis, and sharing of long-term multimodal data from larger patient samples. There is hope that this will allow more representative descriptions of the disorder on a global scale for proper real-world classificatory validations at the subject level. On the verge of providing subject-based information that may assist rational decision-making in the future, for example, as part of “precision boards”—a term analogous to the successful name “tumor boards” currently implemented in multidisciplinary oncology teams—brain connectivity measures can add a unique neurobiological precision component to the evaluation of treatment modalities in complex MDD cases. But first, we need to overcome the existing limitations in the field with consideration of the above-mentioned recommendations to be able to embark on the systems medicine era.

## Data Availability

Data sharing is not applicable to this article as no datasets were generated or analyzed during the current study. The reports of the included and excluded articles are available from the corresponding author upon request.
