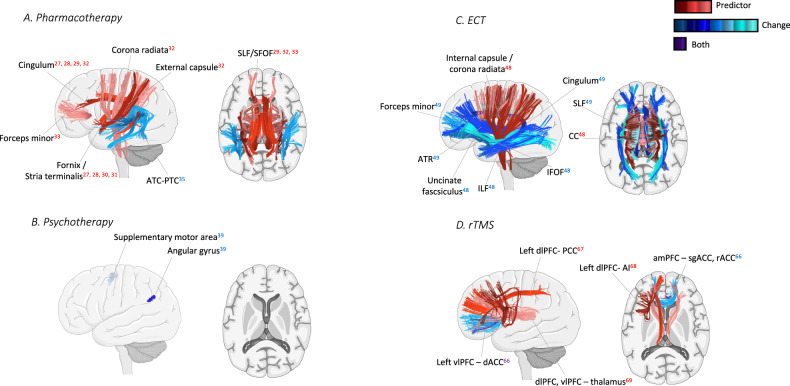# Correction: Brain connectivity in major depressive disorder: a precision component of treatment modalities?

**DOI:** 10.1038/s41398-023-02543-x

**Published:** 2023-07-17

**Authors:** Asude Tura, Roberto Goya-Maldonado

**Affiliations:** Laboratory of Systems Neuroscience and Imaging in Psychiatry (SNIP-Lab), Department of Psychiatry and Psychotherapy, University Medical Center Göttingen (UMG), Göttingen, Germany

**Keywords:** Depression, Predictive markers

Correction to: *Translational Psychiatry* 10.1038/s41398-023-02499-y, published online 09 June 2023

The reference numbers in the Figs. 2 and 3 were given not correct. The figures have been corrected.

Corrected Fig. 2
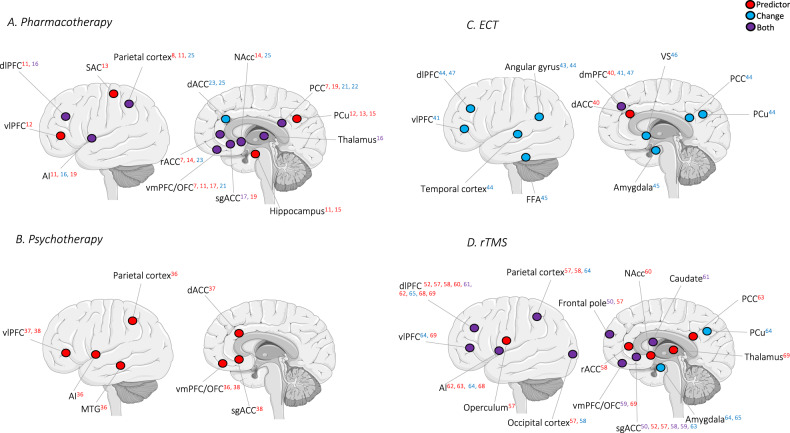


Corrected Fig. 3